# Supplementary material for: Solvent-Free C-3 Coupling of Azaindoles with Cyclic Imines
Source: Molecules. 2019 Oct 4;24(19):3578. doi: 10.3390/molecules24193578 (PMC6803843; doi:10.3390/molecules24193578)
Supplement: Supplementary file 1 [file molecules-24-03578-s001.pdf]

Supplementary Information

Solvent-free C-3 coupling of azaindoles with cyclic imines

Khadija Belasri,[a,b] Ferenc Fülöp[a,b] and István Szatmári,[a,b*]

[a]*Institute of Pharmaceutical Chemistry and* MTA-SZTE Stereochemistry Research Group, *Hungarian Academy of Sciences, University of Szeged, H-6720 Szeged, Eötvös u. 6, Hungary*

Fax: + (36)62545705; E-mail: fulop@pharm.u-szeged.hu

[b]*Institute of Pharmaceutical Chemistry, University of Szeged, Interdisciplinary excellence center*

* *szatmari.istvan@pharm.u-szeged.hu*

3-(1,2,3,4-Tetrahydroisoquinolin-1-yl)-7-azaindole (3).

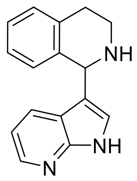

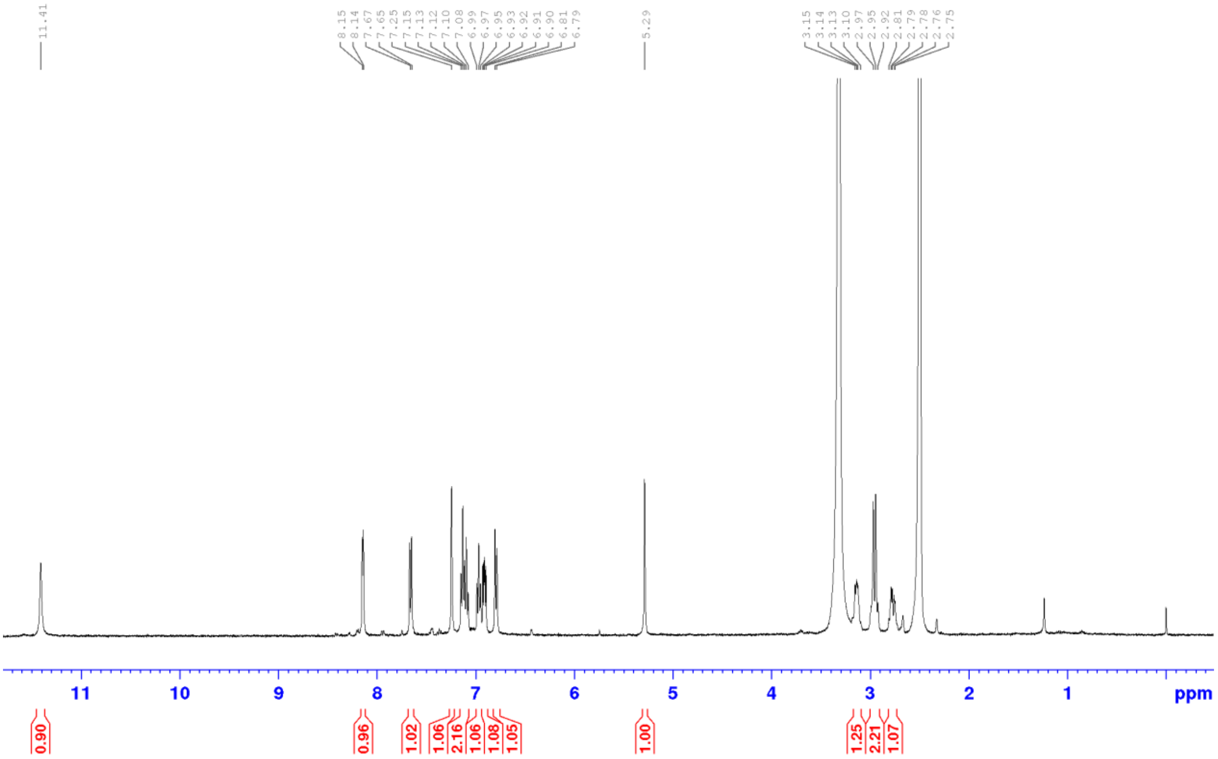

Figure S1. ^{1}H-NMR spectrum of **3**

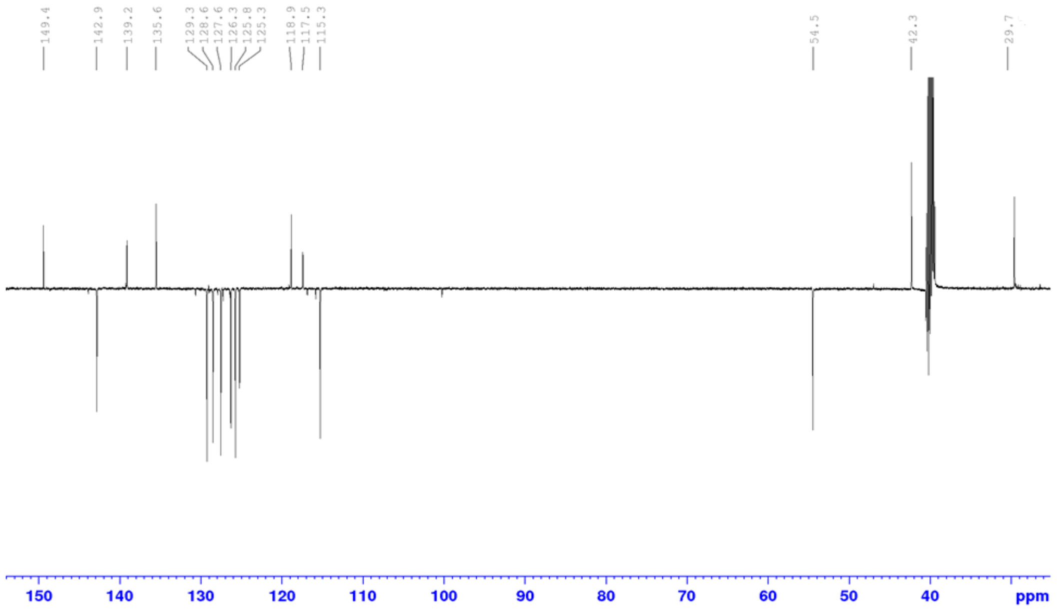

Figure S2. ^{13}C-NMR spectrum of **3**

3-(1,2,3,4-Tetrahydro-*β*-carboline-1-yl)-7-azaindole (7)

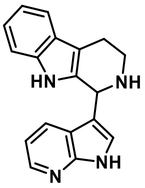

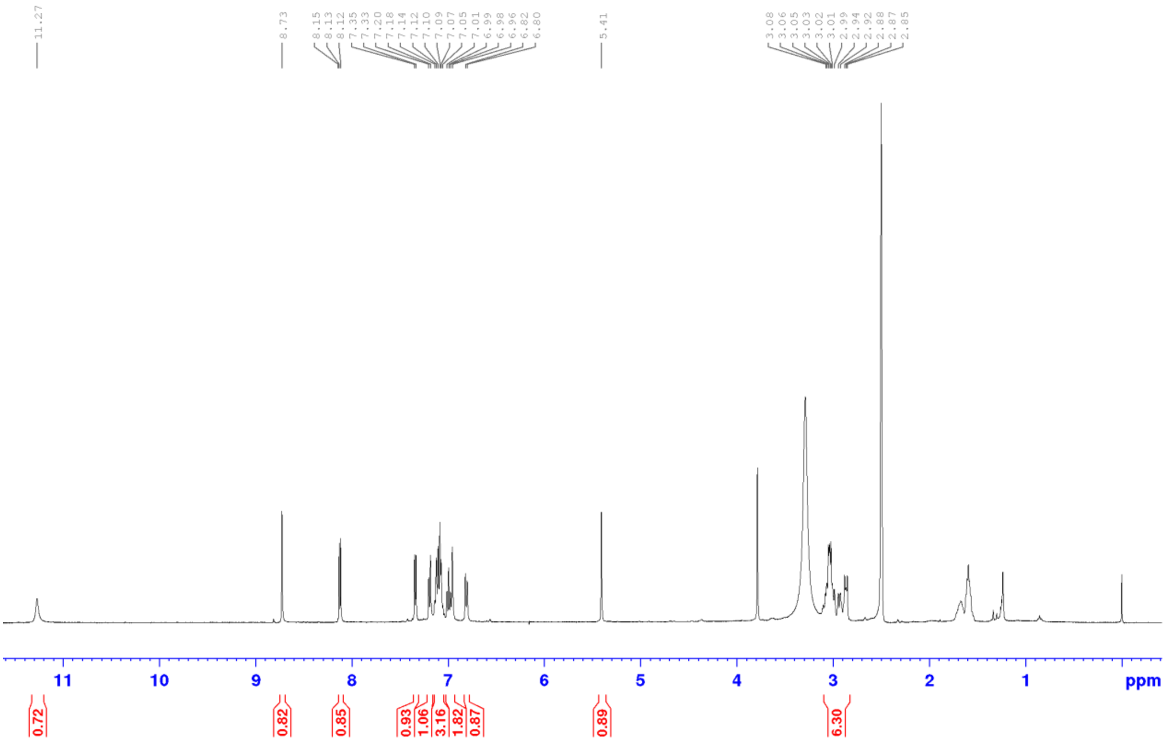

Figure S3. ^{1}H-NMR spectrum of **7**

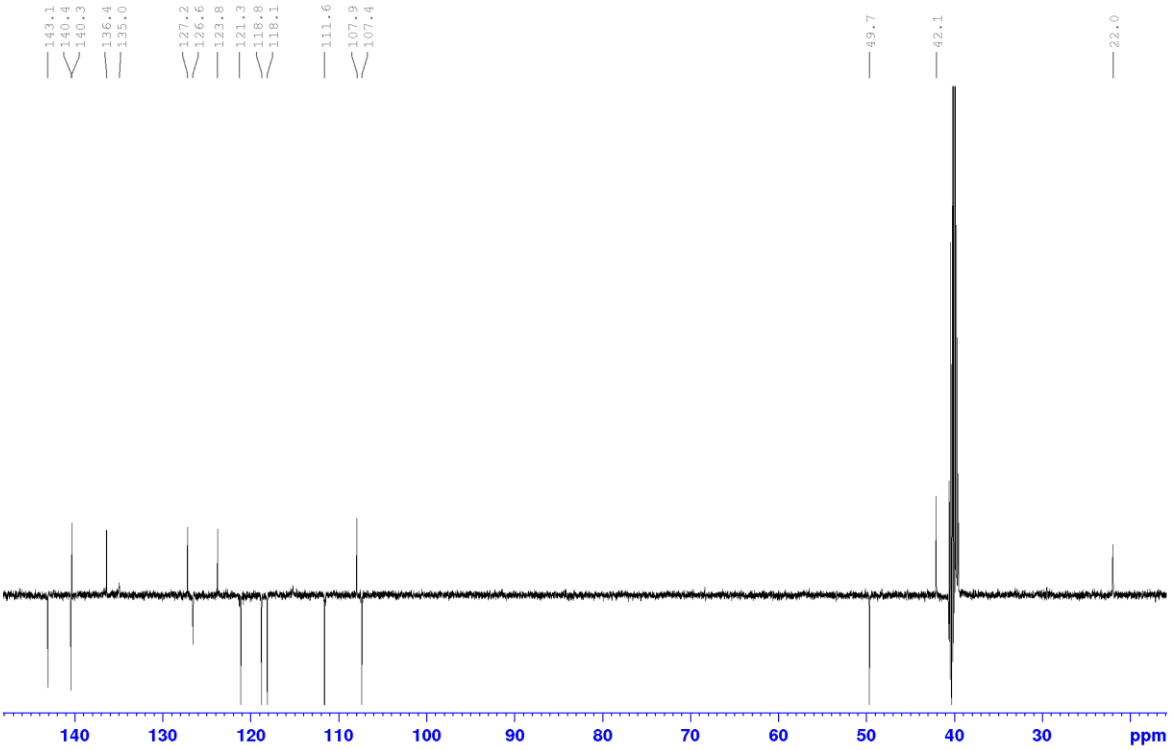

Figure S4. ^{13}C-NMR spectrum of **7**

3-(4,5,6,7-Tetrahydrothieno[3,2-*c*]pyridin-4-yl)-7-azaindole (8)

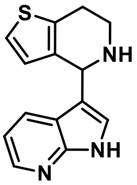

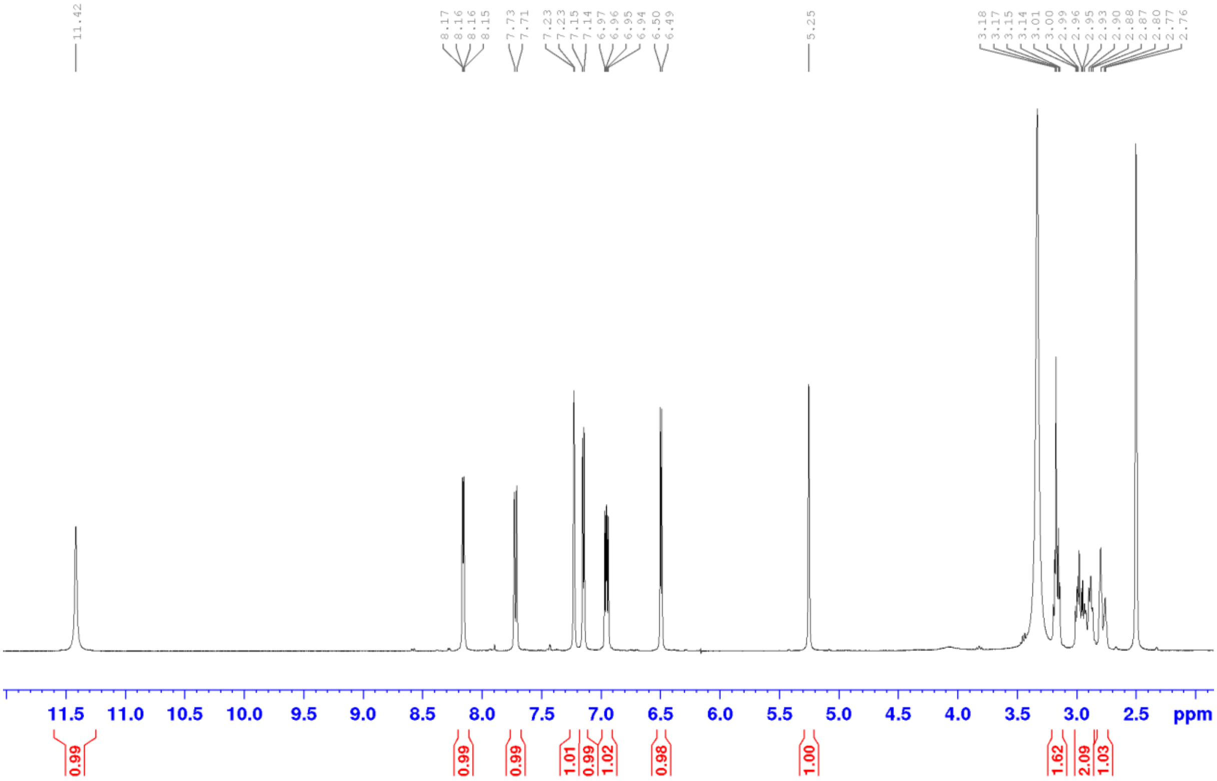

Figure S5. ^{1}H-NMR spectrum of **8**

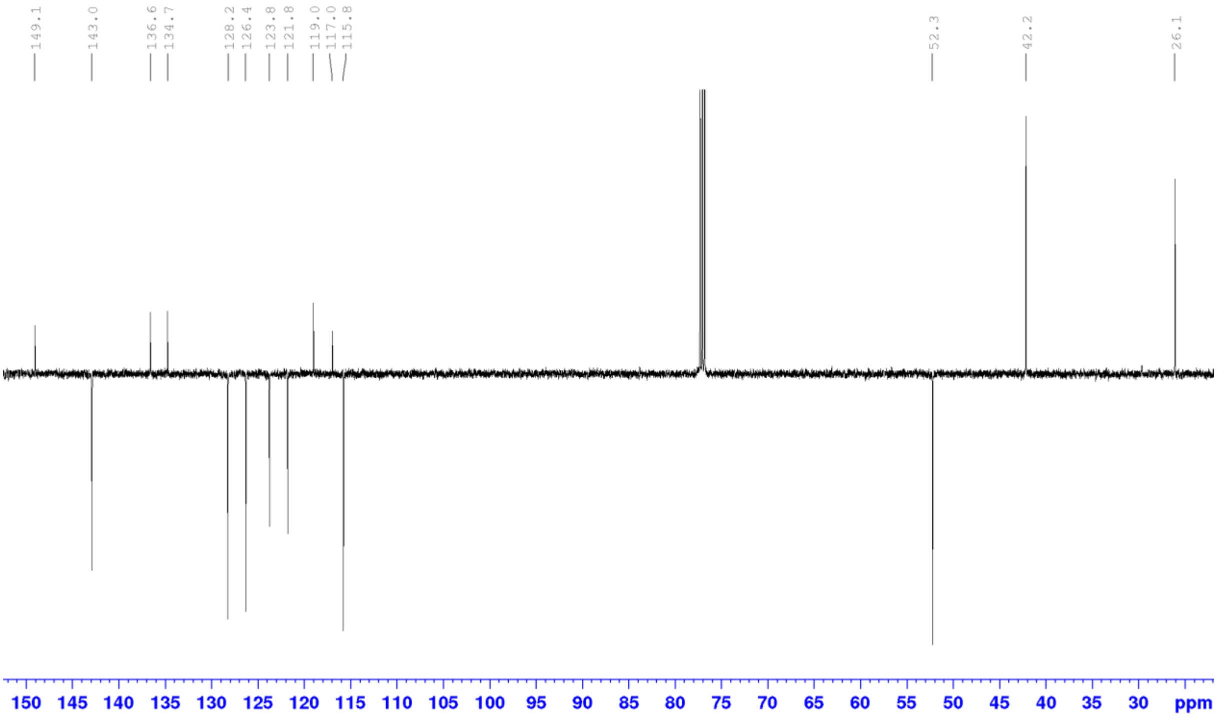

Figure S6. ^{13}C-NMR spectrum of **8**

3-(2,3,4,5-Tetrahydro-*1H*-benz[*c*]azepin-1-yl)-7-azaindole (9)

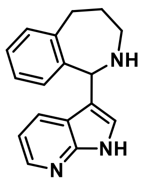

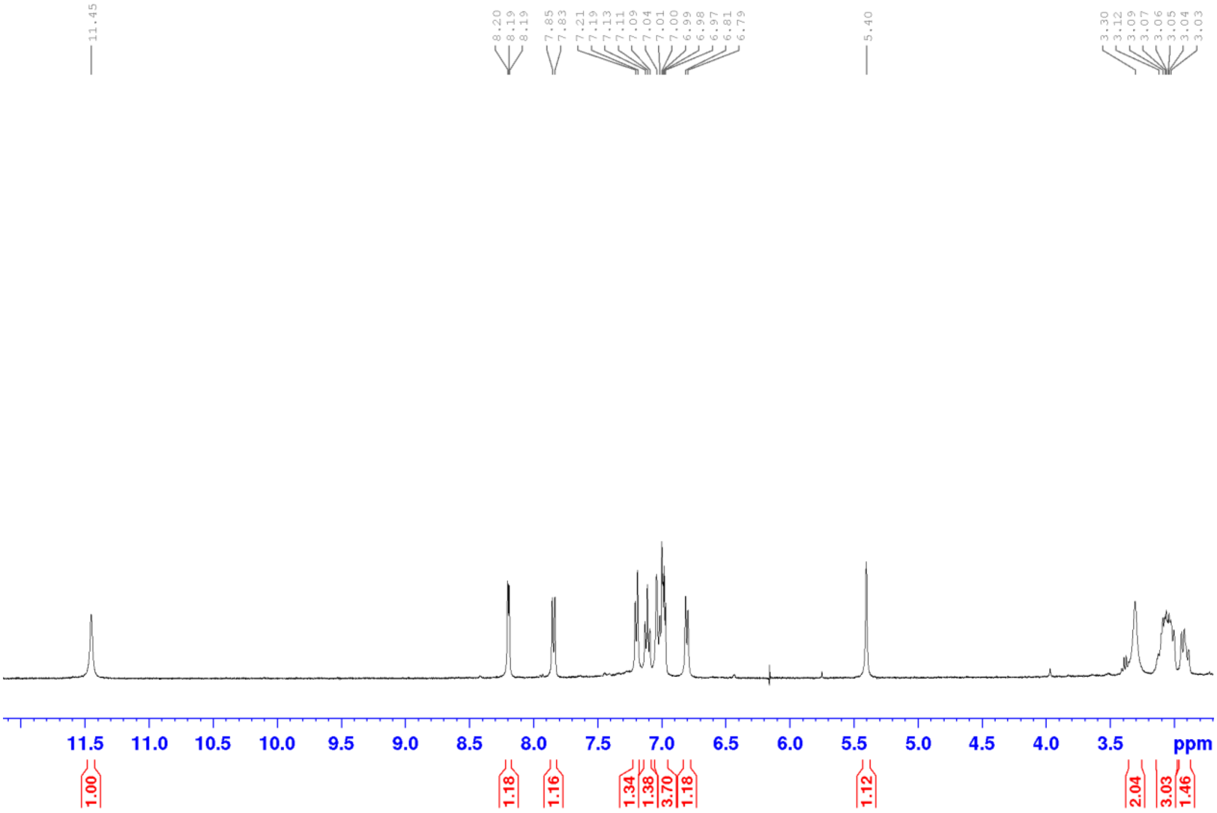

Figure S7. ^{1}H-NMR spectrum of **9**

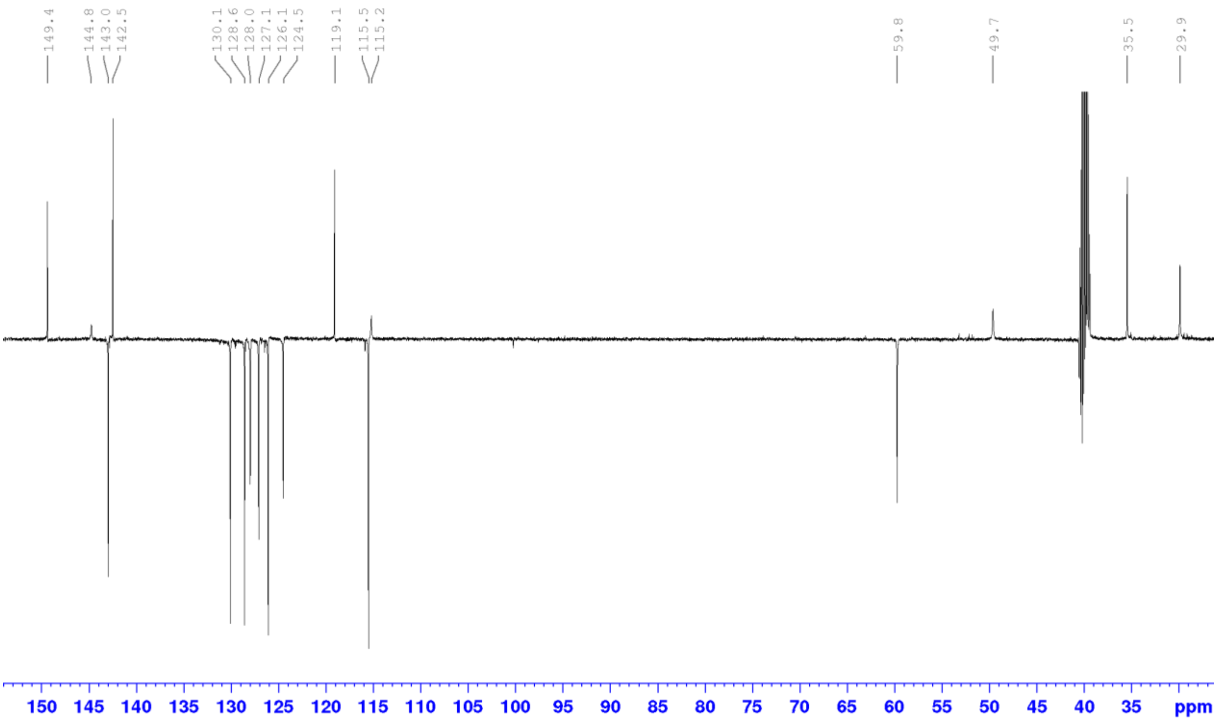

Figure S8. ^{13}C-NMR spectrum of **9**

3-(1,2,3,4-Tetrahydroisoquinolin-1-yl)-4-azaindole (12)

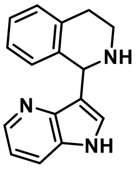

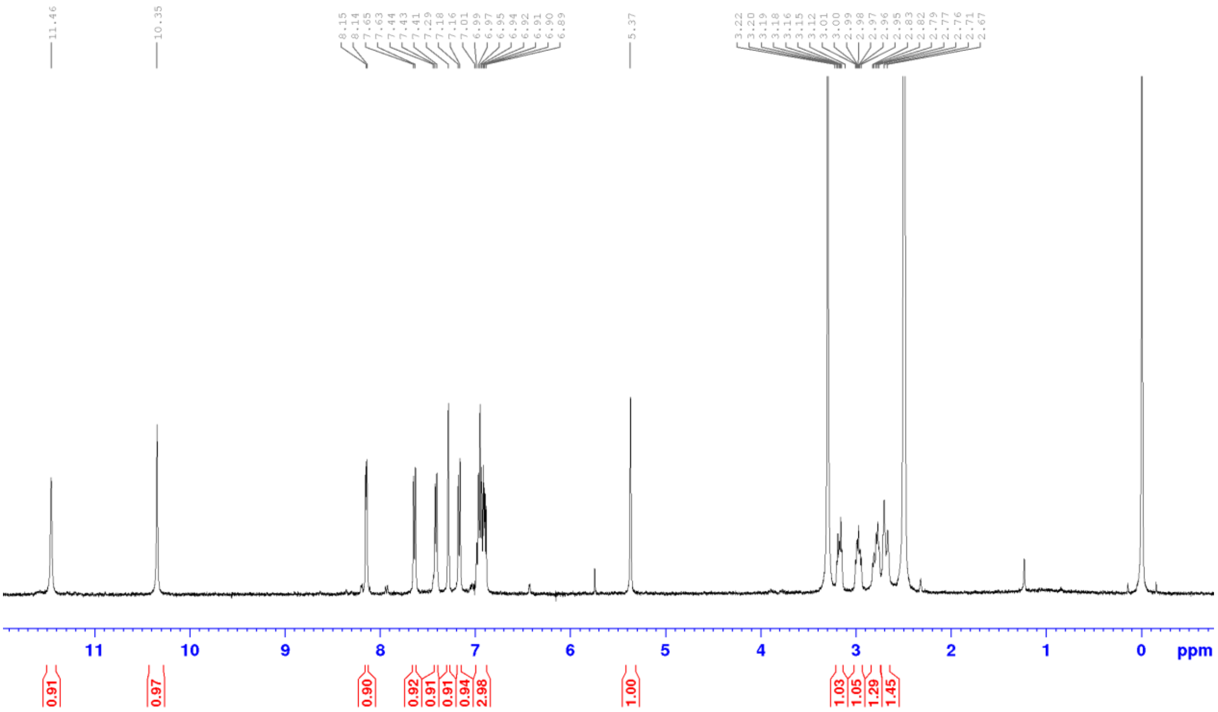

Figure S9. ^{1}H-NMR spectrum of **12**

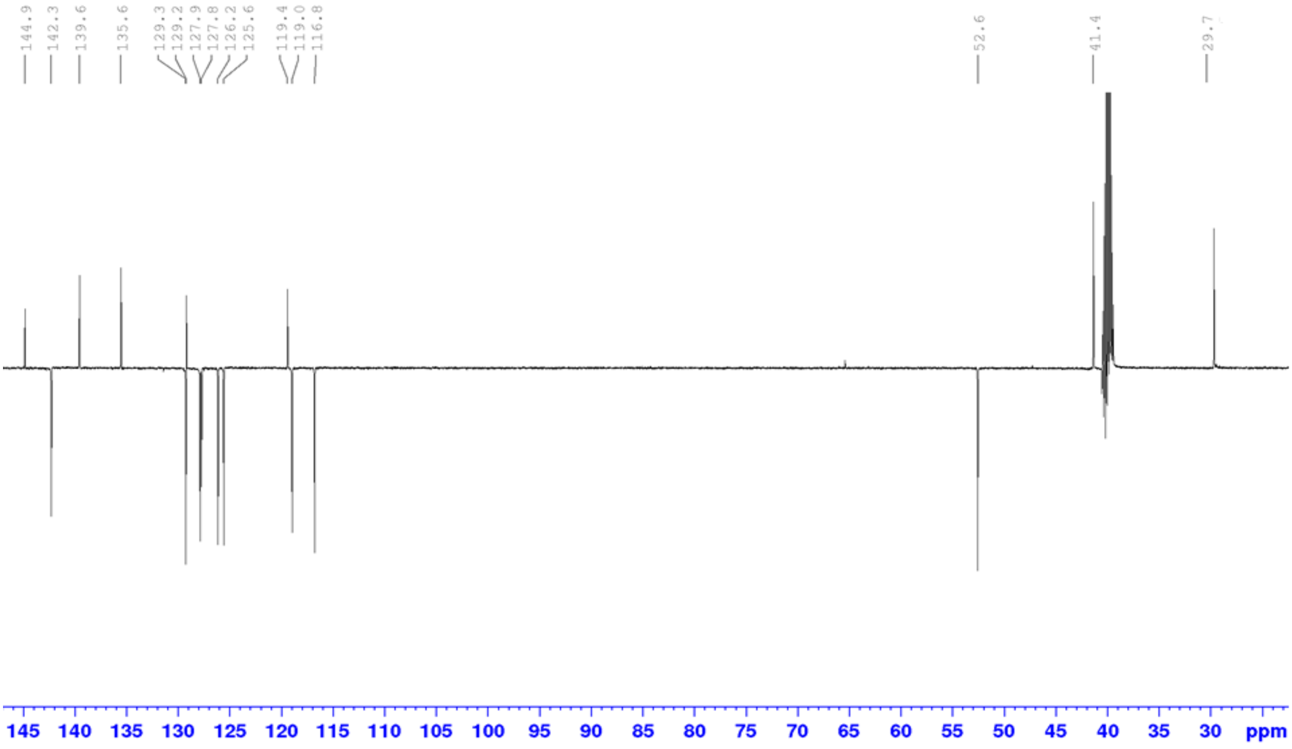

Figure S10. ^{13}C-NMR spectrum of **12**

3-(1,2,3,4-Tetrahydroisoquinolin-1-yl)-6-azaindole (13)

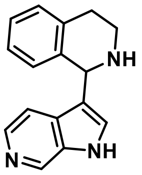

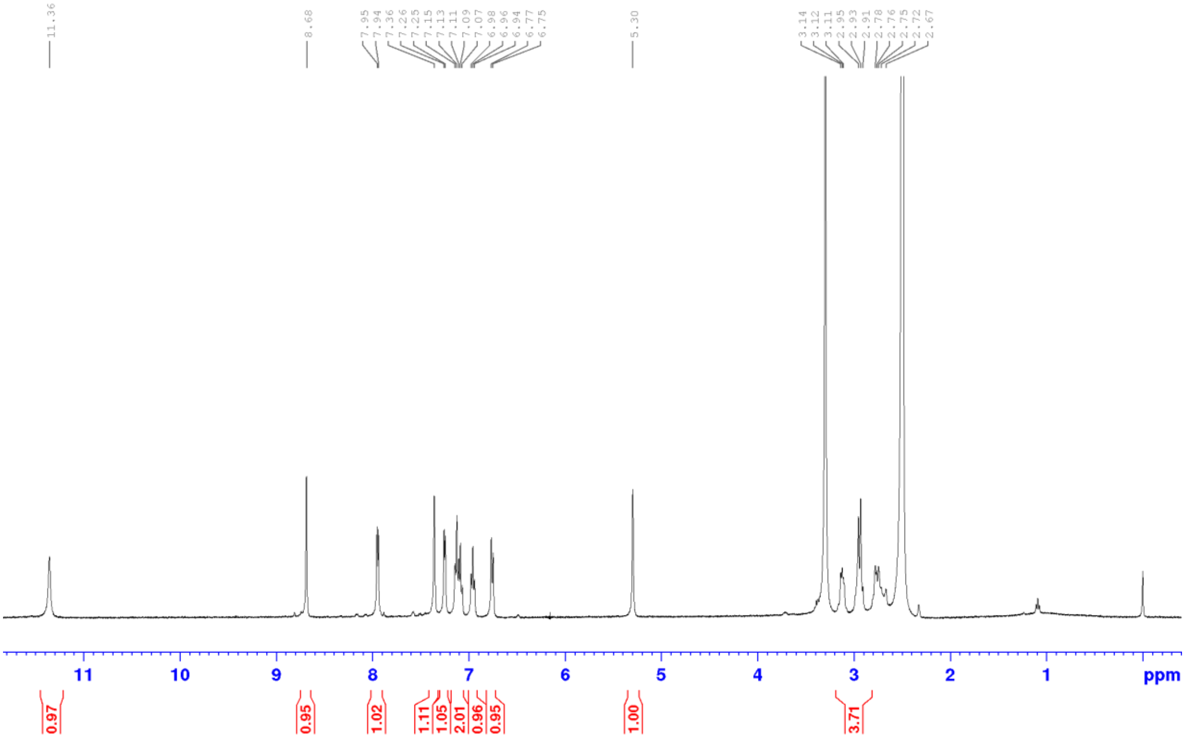

Figure S11. ^{1}H-NMR spectrum of **13**

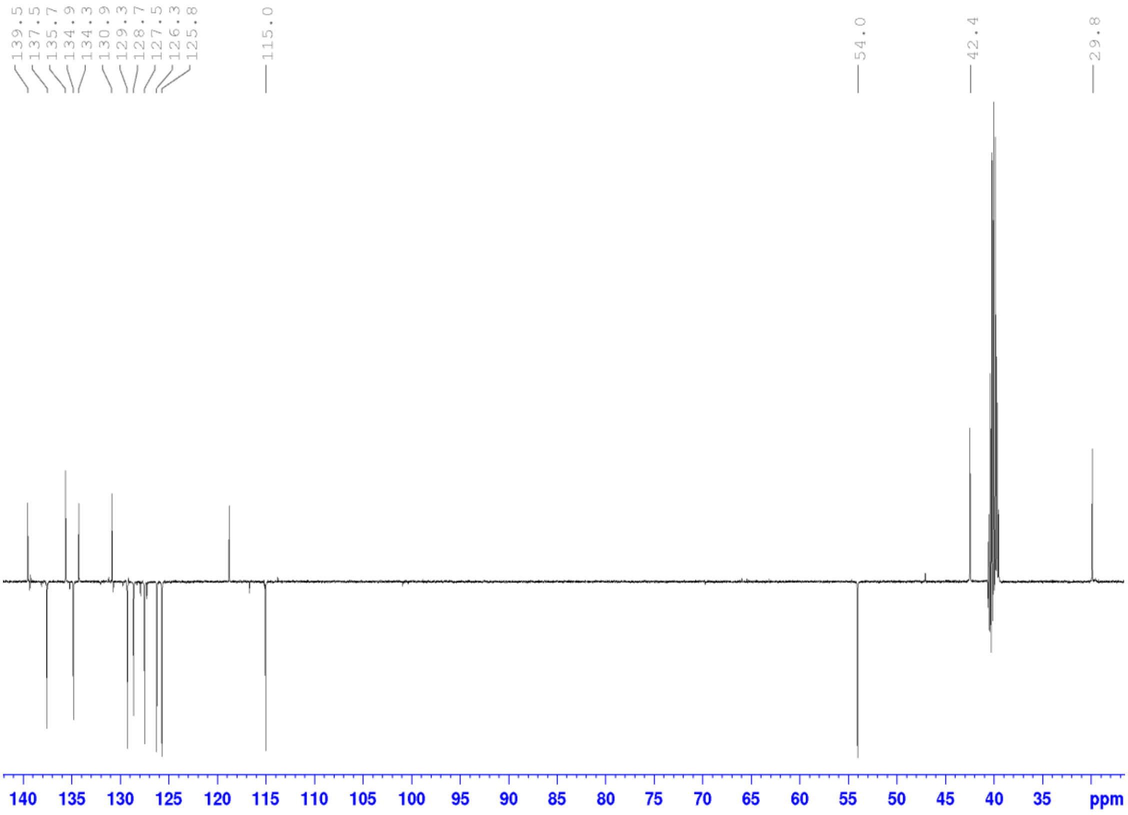

Figure S12. ^{13}C-NMR spectrum of **13**

3-(1,2,3,4-Tetrahydro-*β*-carboline-1-yl)-4-azaindole (14)

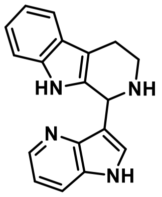

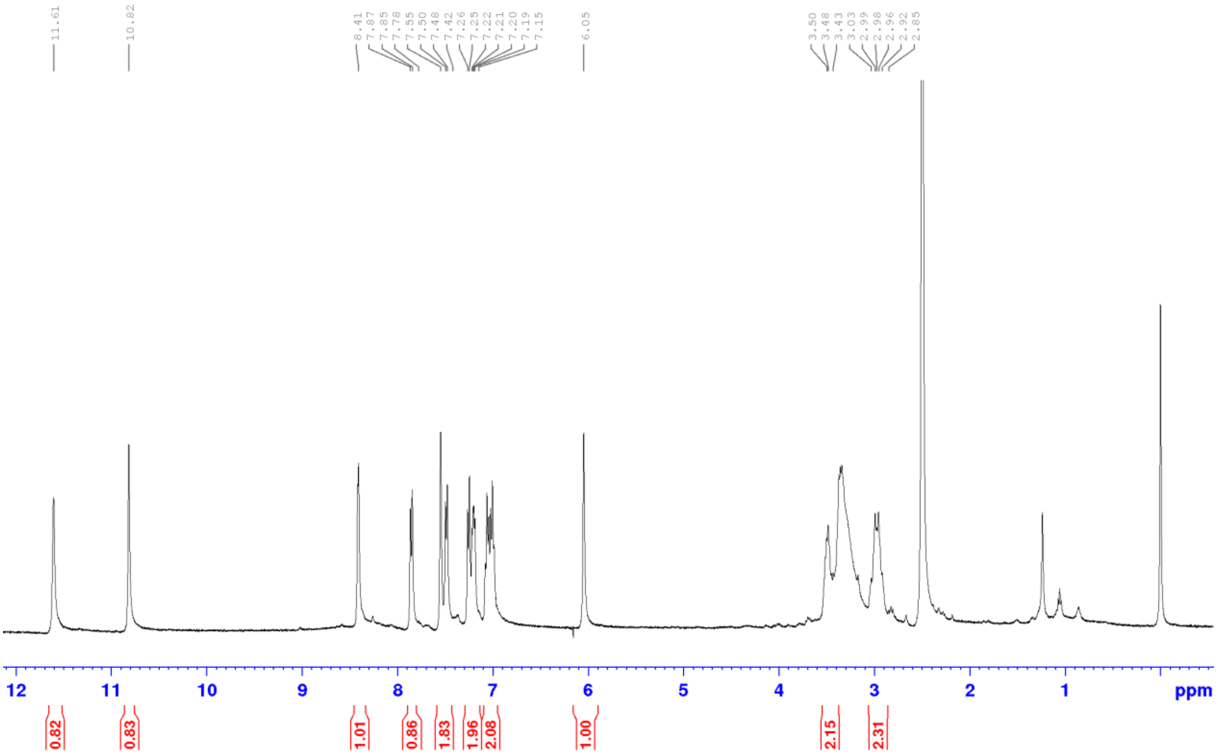

Figure S13. ^{1}H-NMR spectrum of **14**

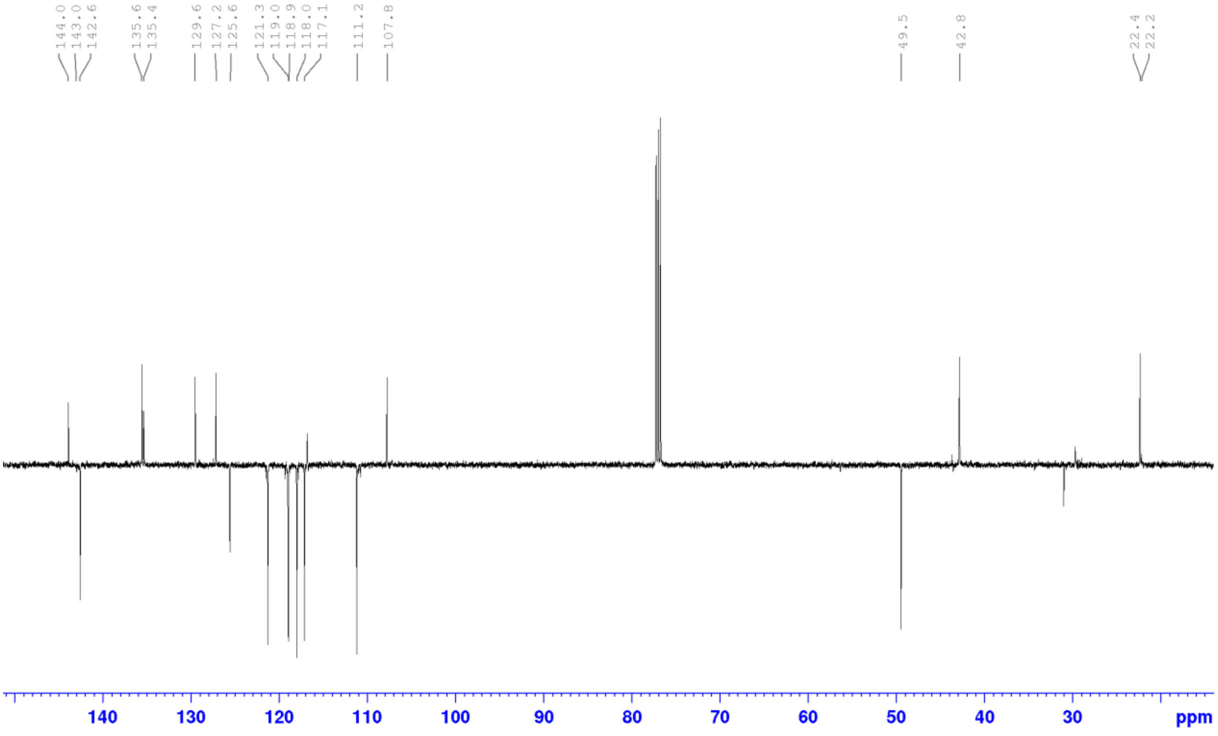

Figure S14. ^{13}C-NMR spectrum of **14**

3-(1,2,3,4-Tetrahydro-β-carboline-1-yl)-6-azaindole (15)

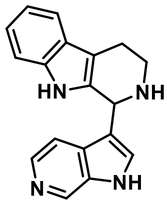

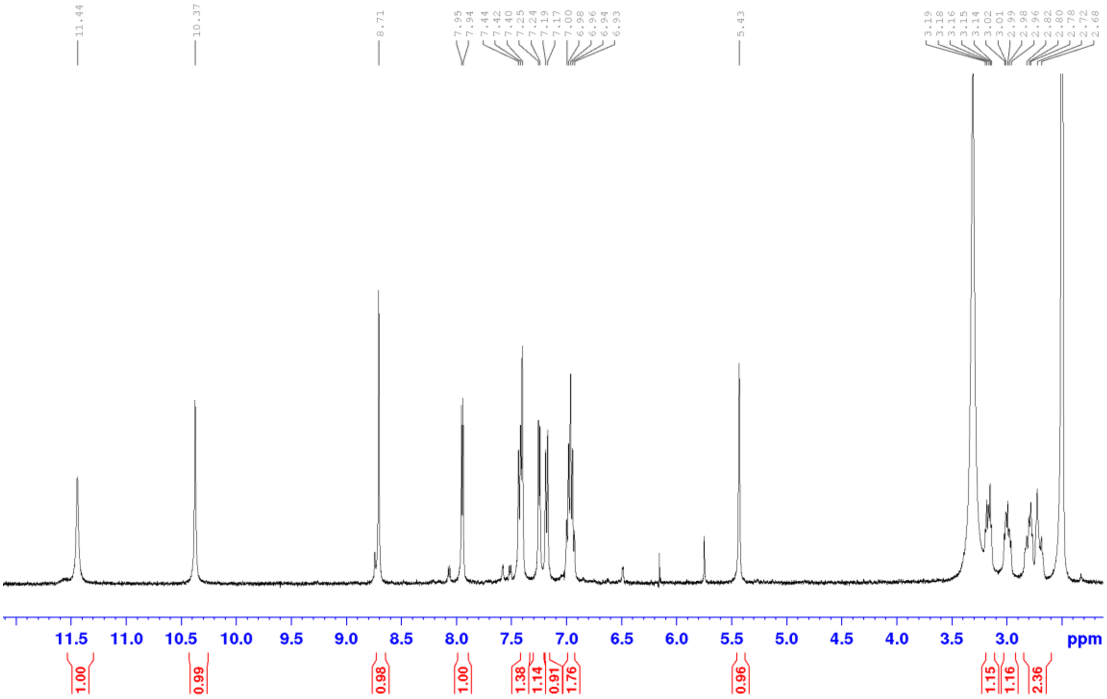

Figure S15. ^{1}H-NMR spectrum of **15**

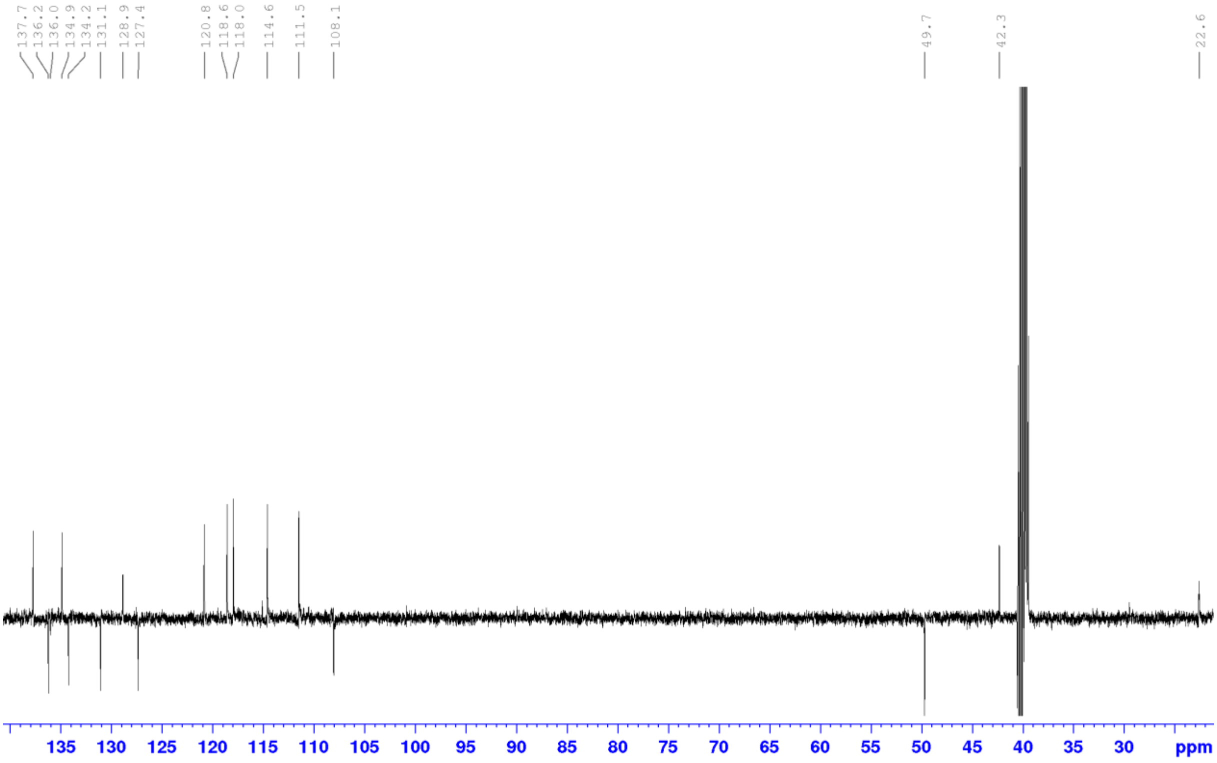

Figure S16. ^{13}C-NMR spectrum of **15**

3-(4,5,6,7-Tetrahydrothieno[3,2-*c*]pyridin-4-yl)-4-azaindole (16)

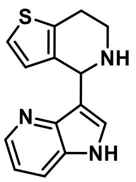

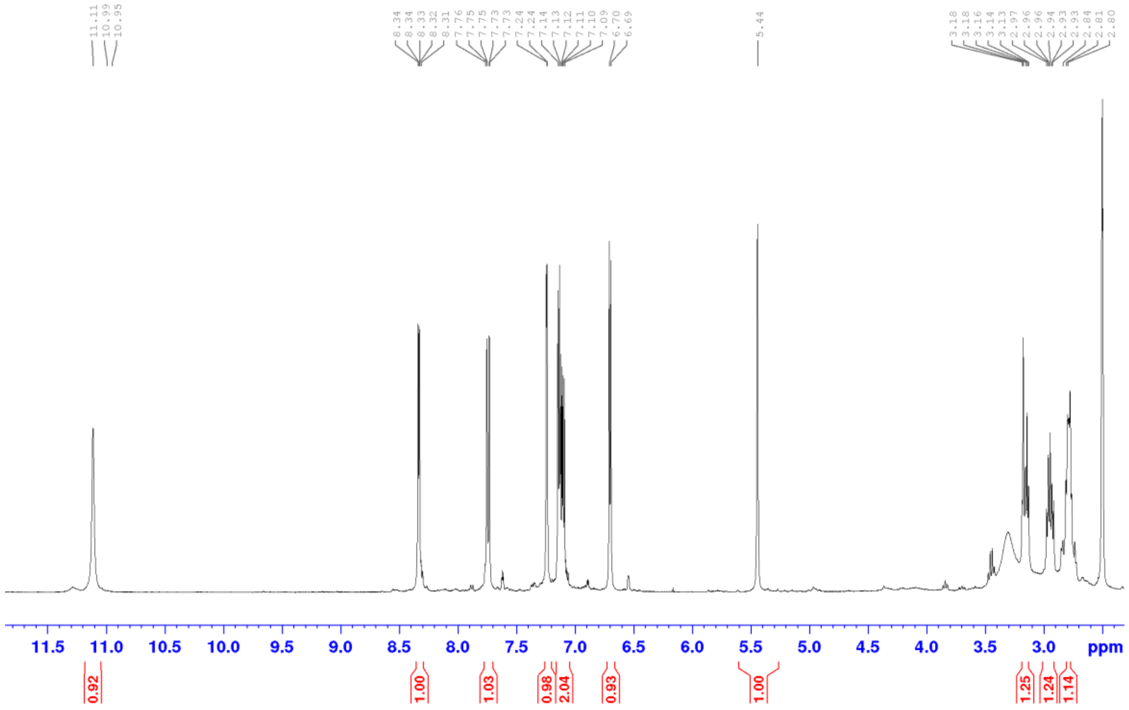

Figure S17. ^{1}H-NMR spectrum of **16**

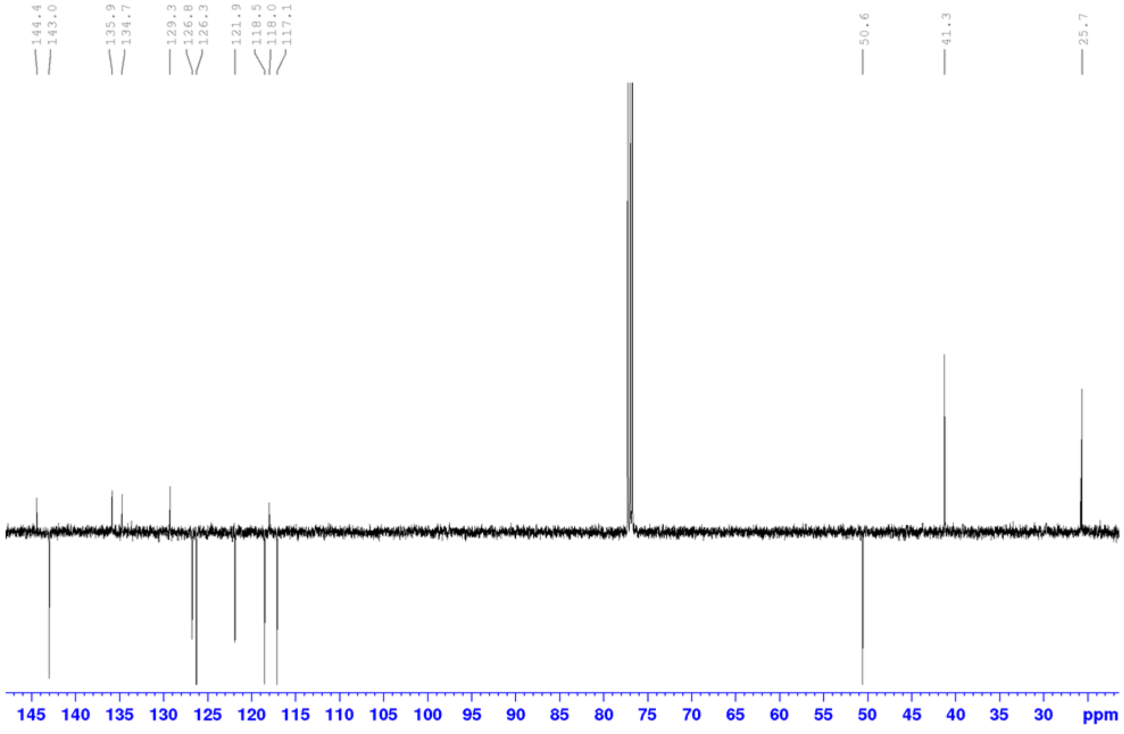

Figure S18. ^{13}C-NMR spectrum of **16**

3-(4,5,6,7-Tetrahydrothieno[3,2-*c*]pyridin-4-yl)-6-azaindole (17)

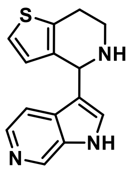

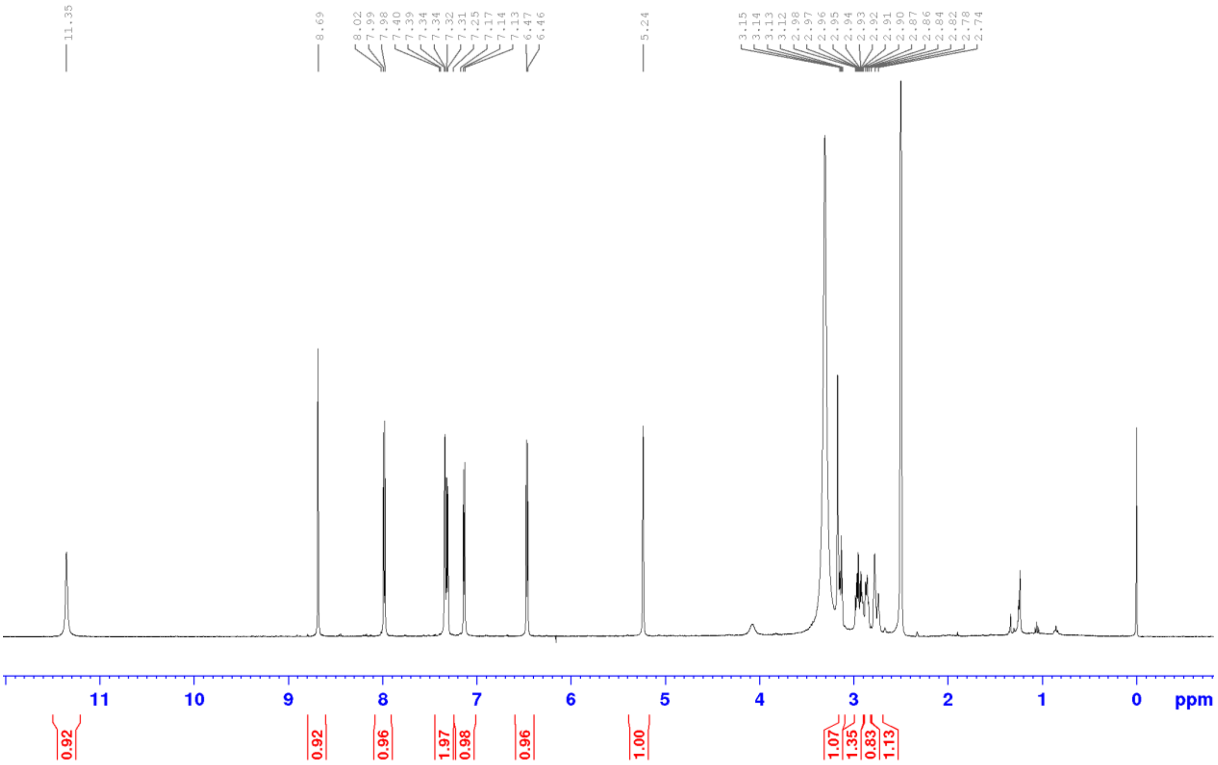

Figure S19. ^{1}H-NMR spectrum of **17**

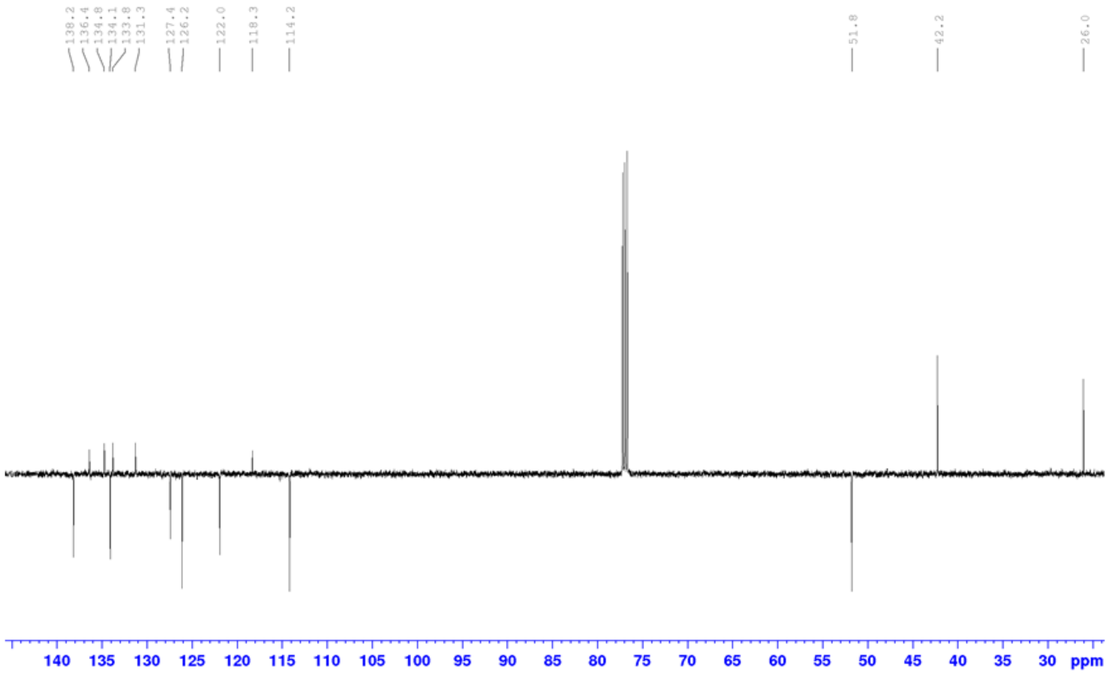

Figure S20. ^{13}C-NMR spectrum of **17**

3-(2,3,4,5-Tetrahydro-*1H*-benz[*c*]azepin-1-yl)-4-azaindole (18)

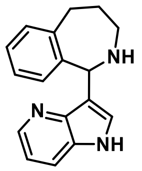

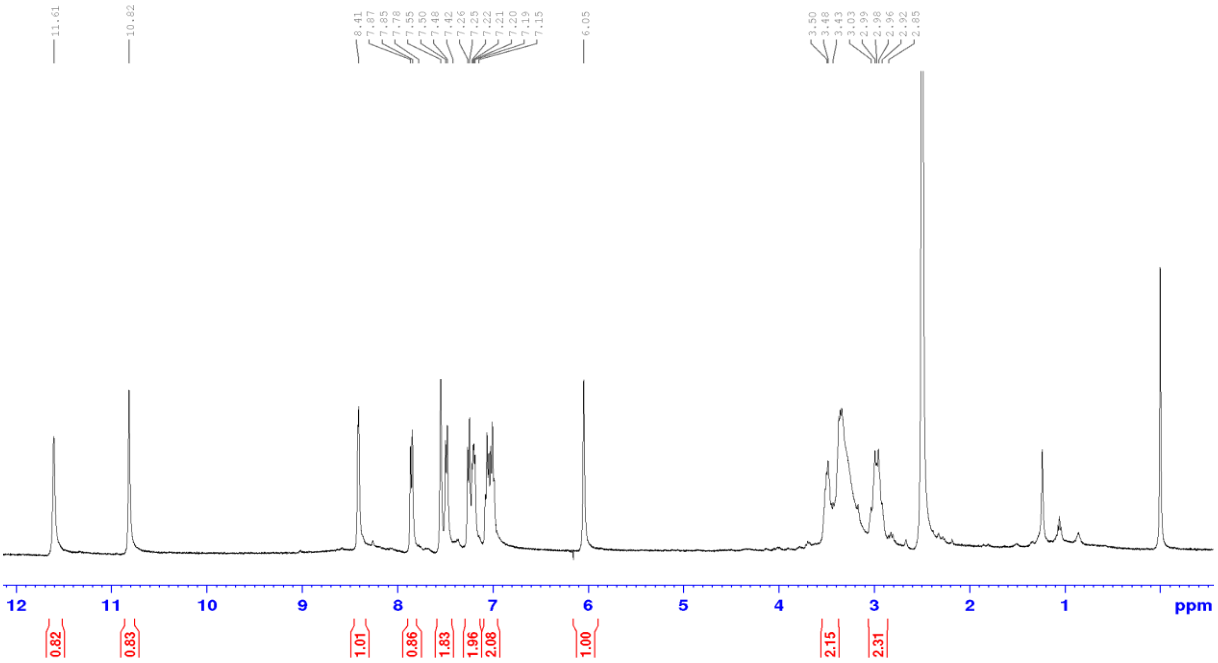

Figure S21. ^{1}H-NMR spectrum of **18**

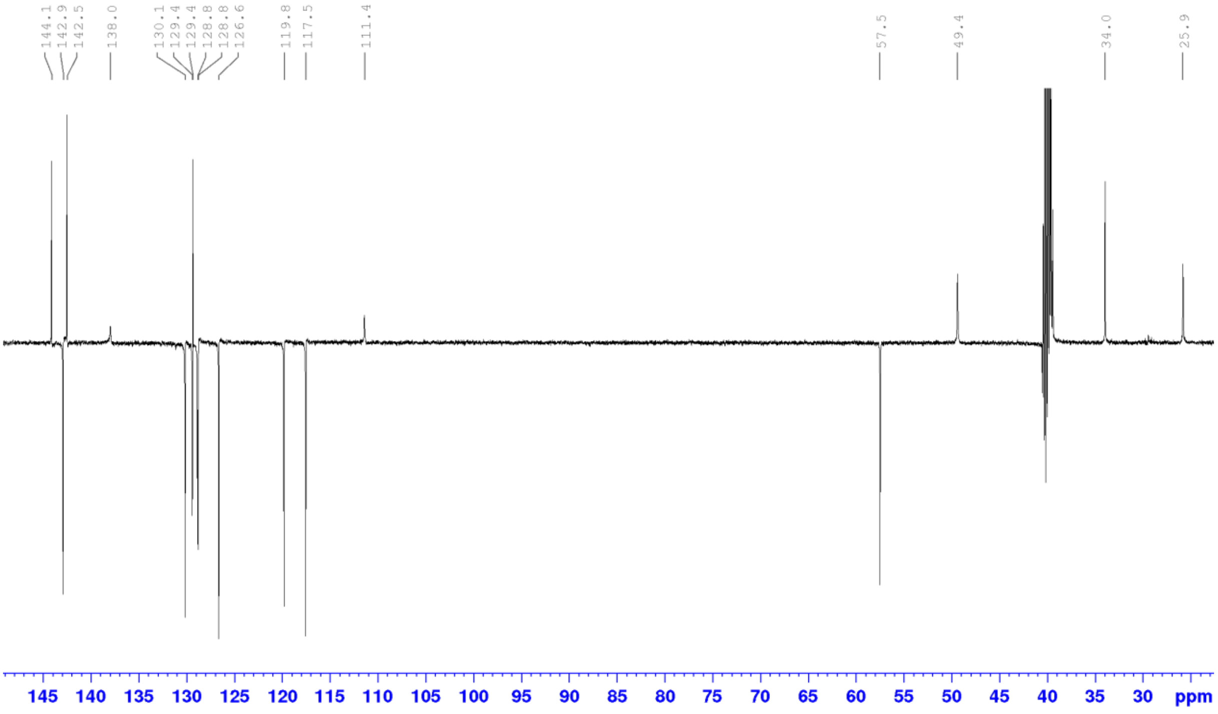

Figure S22. ^{13}C-NMR spectrum of **18**

3-(2,3,4,5-Tetrahydro-*1H*-benz[*c*]azepin-1-yl)-6-azaindole (19)

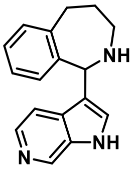

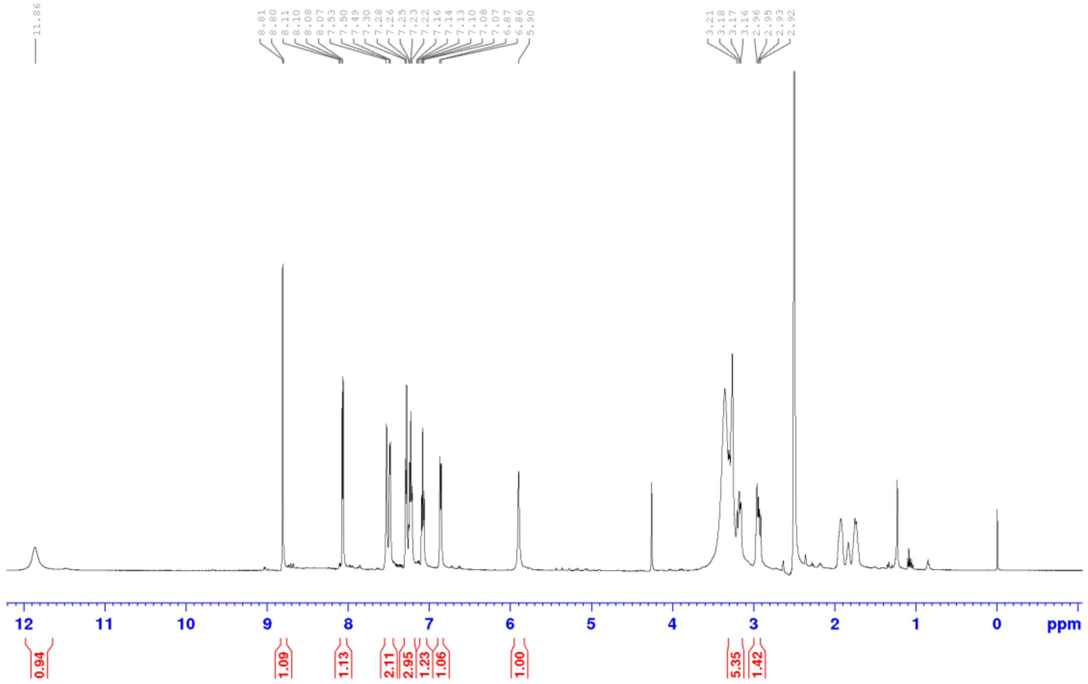

Figure S23. ^{1}H-NMR spectrum of **19**

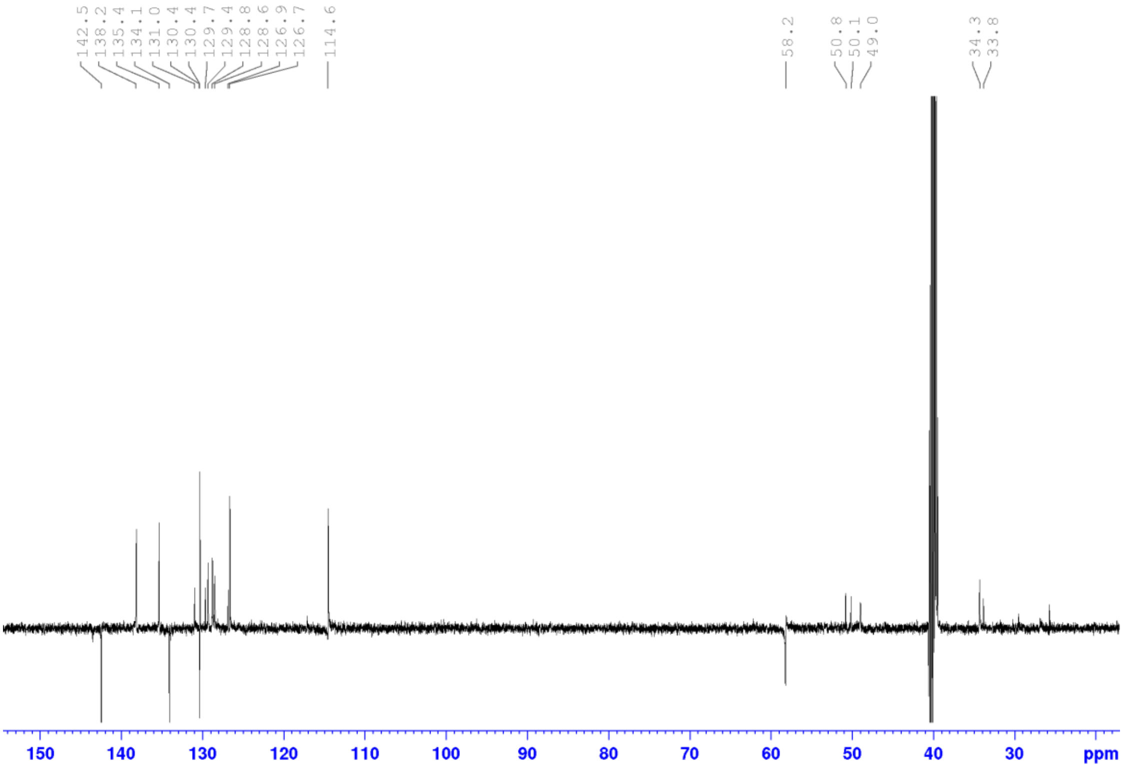

Figure S24. ^{13}C-NMR spectrum of **19**

3-(1,2,3,4-Tetrahydroisoquinolin-1-yl)-5-azaindole (21)

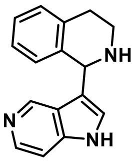

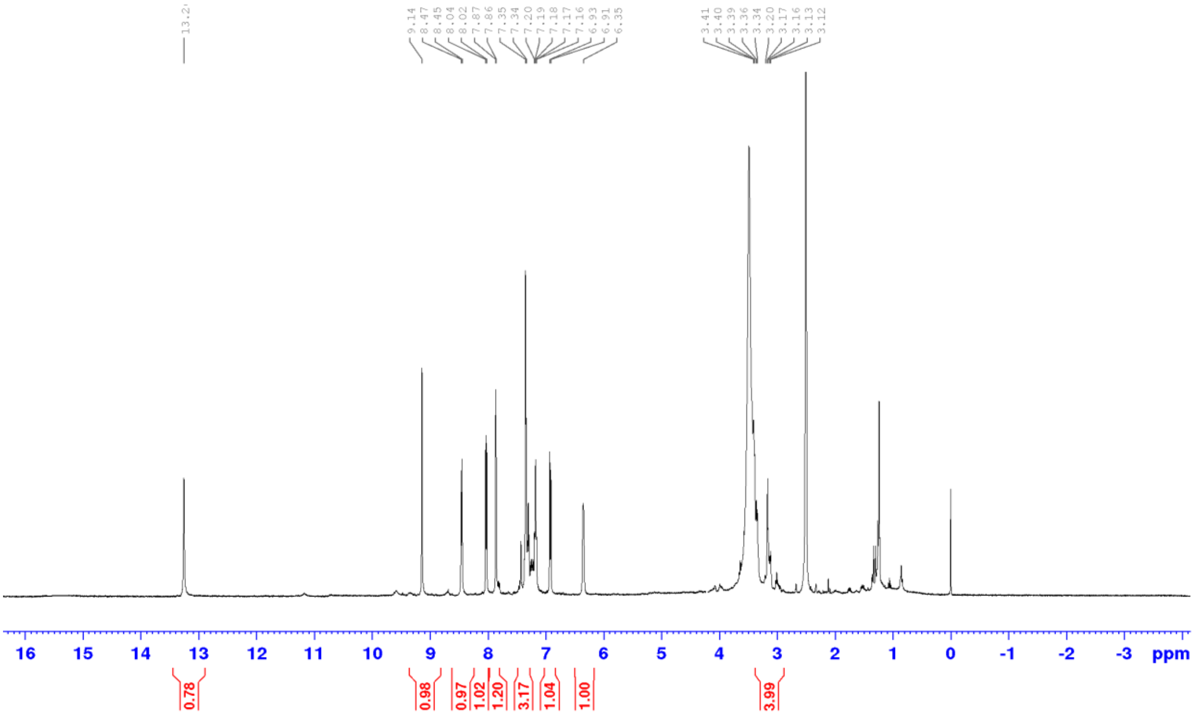

Figure S25. ^{1}H-NMR spectrum of **21**

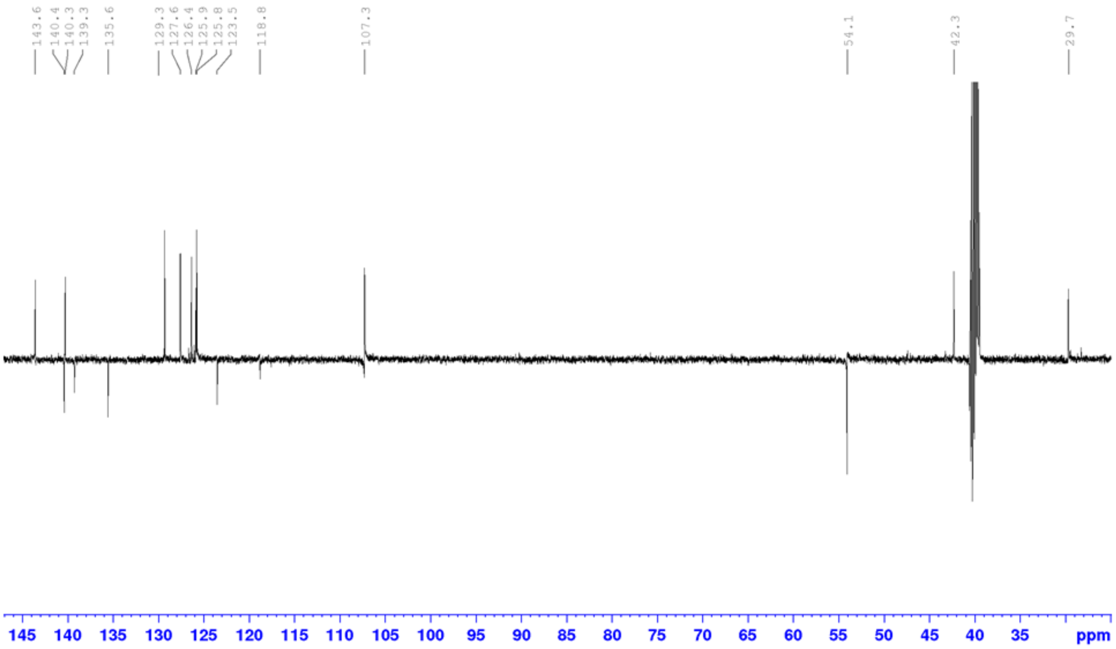

Figure S26. ^{13}C-NMR spectrum of **21**

3-(1,2,3,4-Tetrahydro-*β*-carboline-1-yl)-5-azaindole (22)

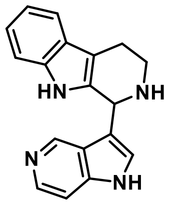

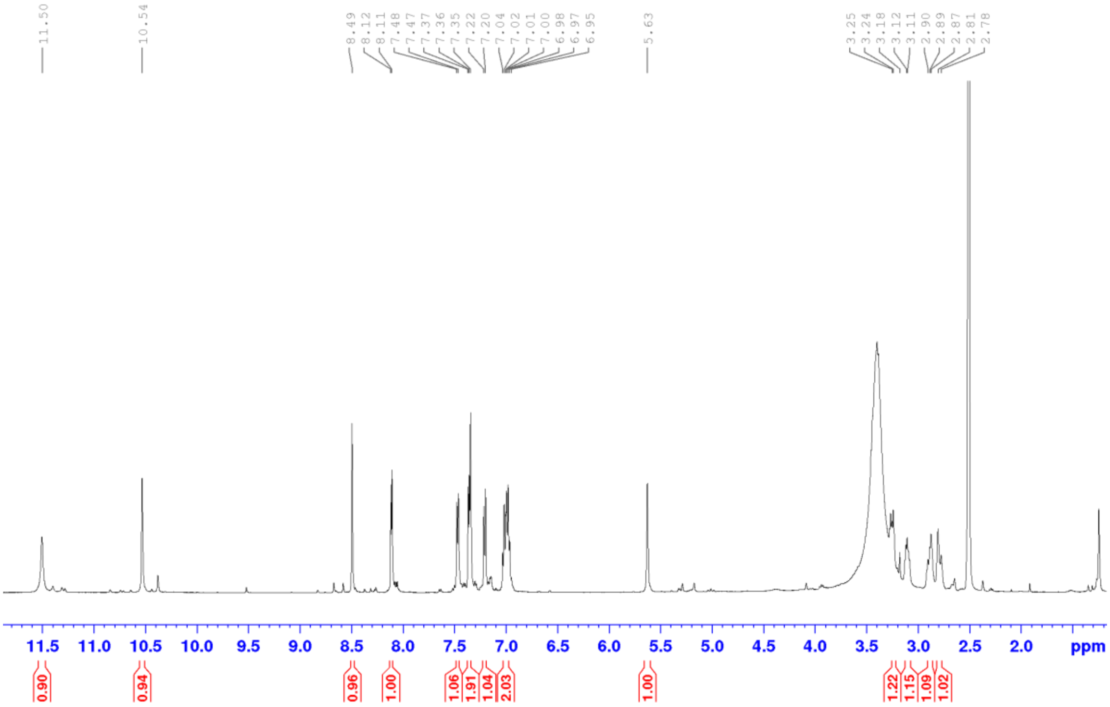

Figure S27. ^{1}H-NMR spectrum of **22**

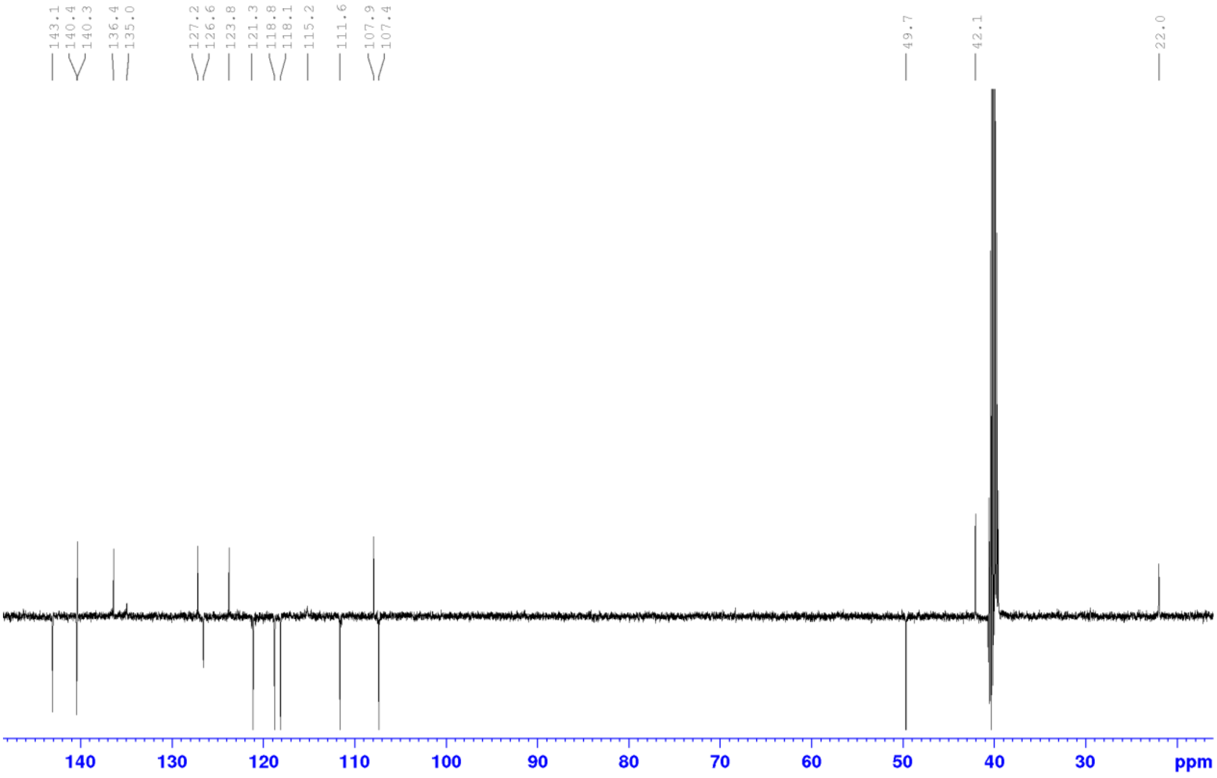

Figure S28. ^{13}C-NMR spectrum of **22**

3-(4,5,6,7-Tetrahydrothieno[3,2-*c*]pyridin-4-yl)-5-azaindole (23)

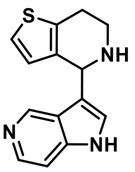

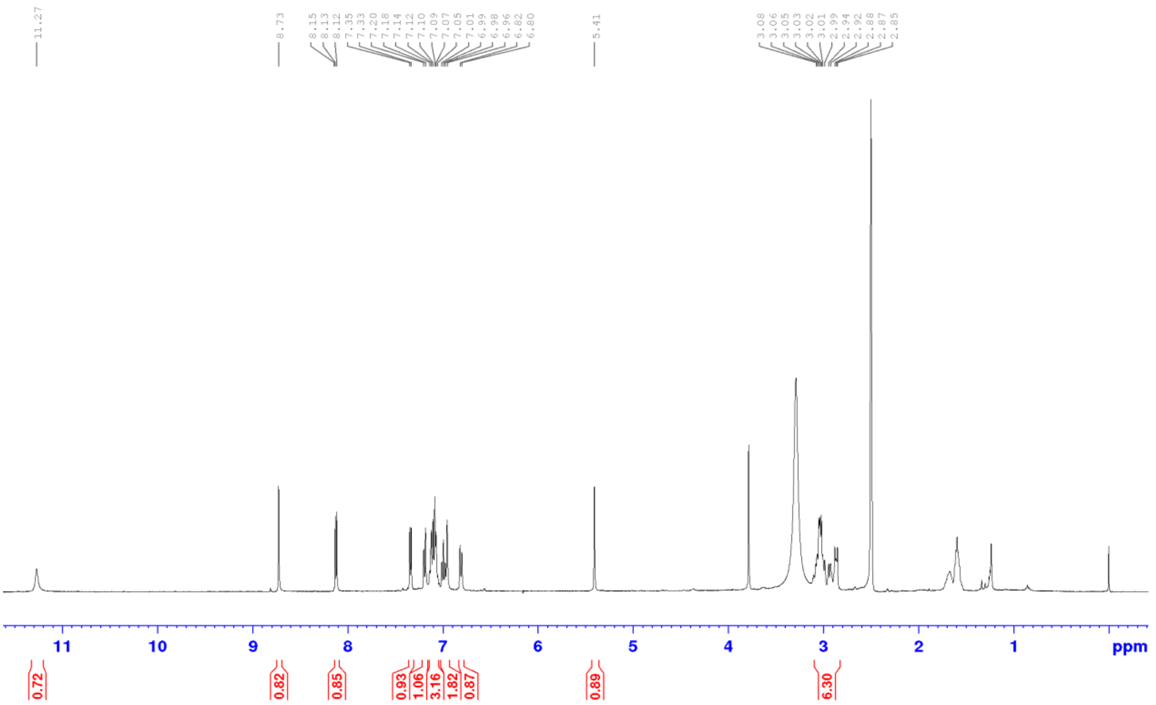

Figure S29. ^{1}H-NMR spectrum of **23**

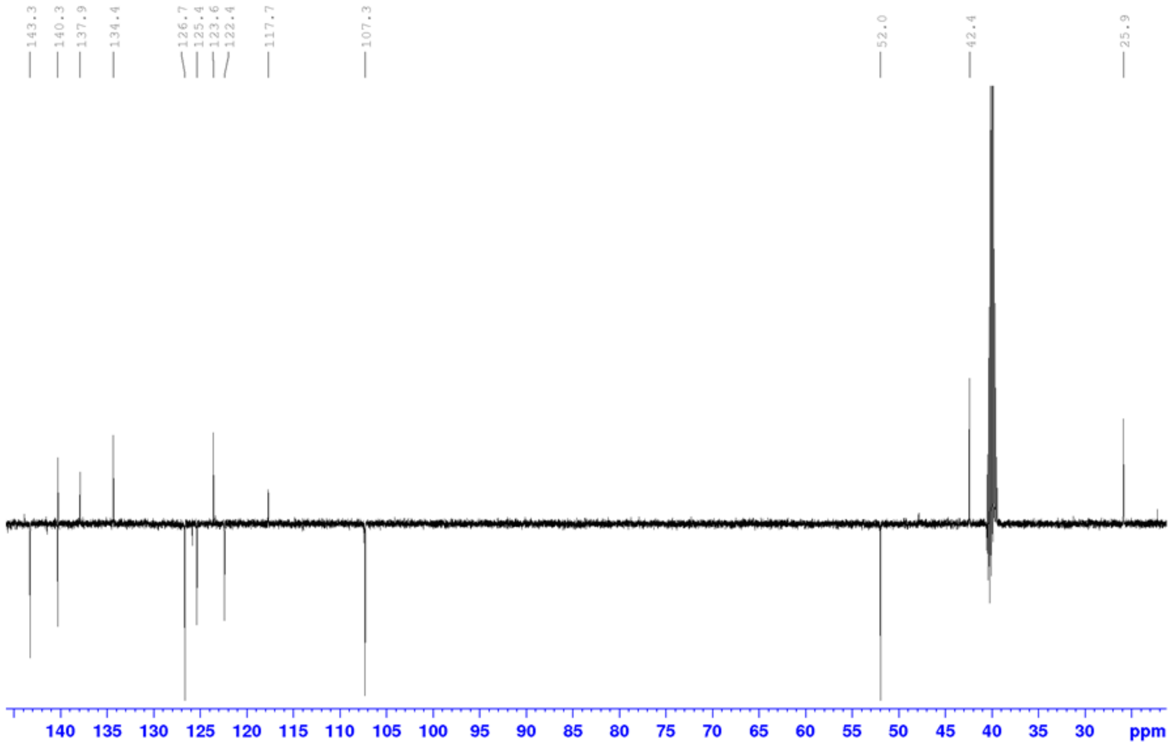

Figure S30. ^{13}C-NMR spectrum of **23**

3-(2,3,4,5-Tetrahydro-*1H*-benzo[*c*]azepin-1-yl)-5-azaindole (24)

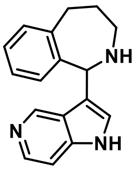

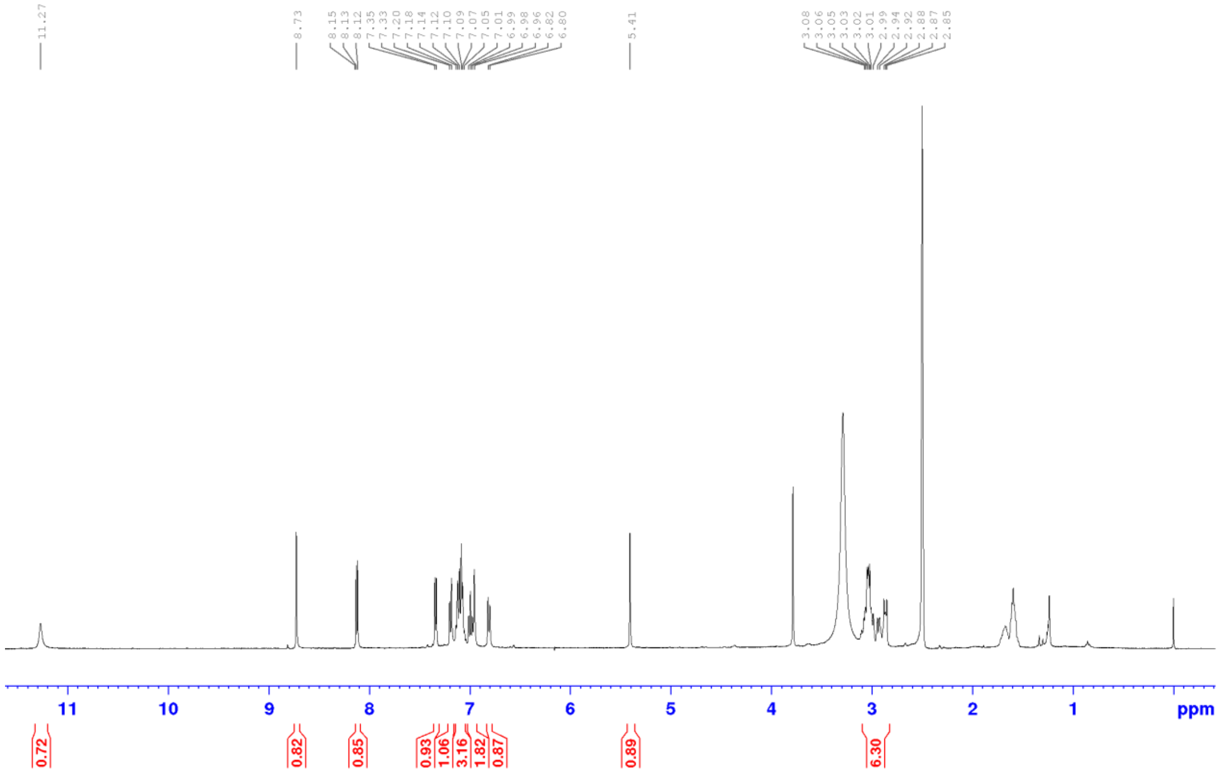

Figure S31. ^{1}H-NMR spectrum of **24**

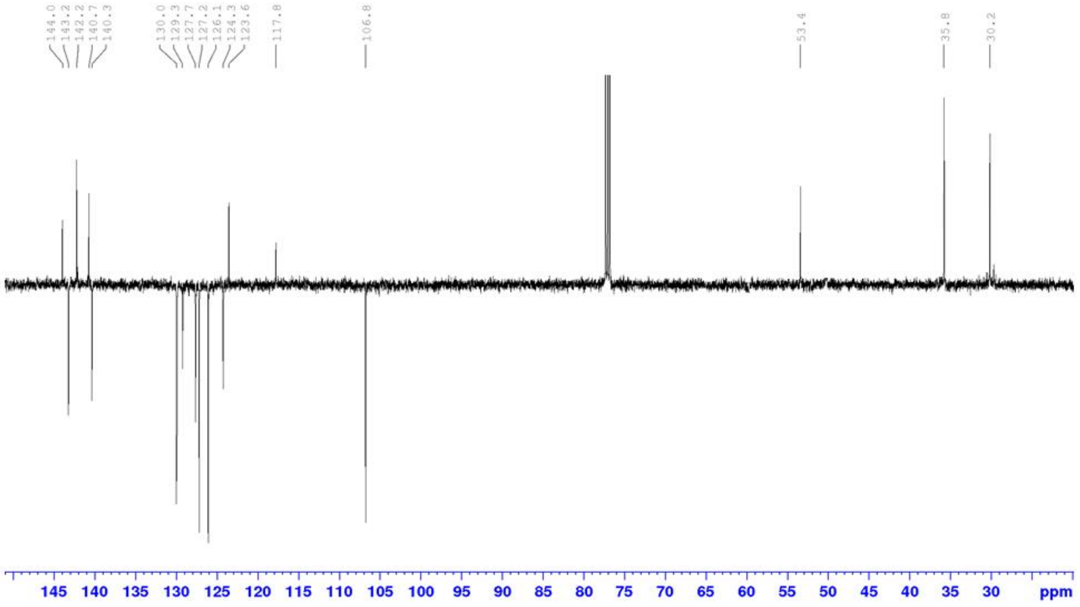

Figure S32. ^{13}C-NMR spectrum of **24**
